# Isolation and characterization of stromal progenitor cells from ascites of patients with epithelial ovarian adenocarcinoma

**DOI:** 10.1186/1423-0127-19-23

**Published:** 2012-02-14

**Authors:** Chih-Ming Ho, Shwu-Fen Chang, Chih-Chiang Hsiao, Tsai-Yen Chien, Daniel Tzu-Bi Shih

**Affiliations:** 1 Gynecologic Cancer Center, Department of Obstetrics and Gynecology, Cathay General Hospital, Taipei, Taiwan; 2 School of Medicine, Fu Jen Catholic University, Hsinchuang, Taipei Hsien, Taiwan; 3 School of Medicine, Taipei Medical University, Taipei, Taiwan; 4 Department of Medical Research, Cathay General Hospital, Sijhih, Taipei Hsien, Taiwan; 5 Graduate Institute of Medical Sciences, School of Medicine, Taipei Medical University, Taipei, Taiwan

**Keywords:** human cancer initiating/stem cell, stromal progenitor cells, epithelial ovarian adenocarcinoma, epithelial-mesenchymal transition

## Abstract

**Background:**

At least one-third of epithelial ovarian cancers are associated with the development of ascites containing heterogeneous cell populations, including tumor cells, inflammatory cells, and stromal elements. The components of ascites and their effects on the tumor cell microenvironment remain poorly understood. This study aimed to isolate and characterize stromal progenitor cells from the ascites of patients with epithelial ovarian adenocarcinoma (EOA).

**Methods:**

Seventeen ascitic fluid samples and 7 fresh tissue samples were collected from 16 patients with EOA. The ascites samples were then cultured in vitro in varying conditions. Flow cytometry and immunocytochemistry were used to isolate and characterize 2 cell populations with different morphologies (epithelial type and mesenchymal type) deriving from the ascites samples. The in vitro cell culture model was established using conditional culture medium.

**Results:**

The doubling times of the epithelial type and mesenchymal type cells were 36 h and 48 h, respectively, indicating faster growth of the epithelial type cells compared to the mesenchymal type cells. Cultured in vitro, these ascitic cells displayed the potential for self-renewal and long-term proliferation, and expressed the typical cancer stem/progenitor cell markers CD44^high^, CD24^low^, and AC133^+^. These cells also demonstrated high BMP-2, BMP4, TGF-β, Rex-1, and AC133 early gene expression, and expressed EGFR, integrin α_2_β_1_, CD146, and Flt-4, which are highly associated with tumorigenesis and metastasis. The epithelial type cells demonstrated higher cytokeratin 18 and E-cadherin expression than the mesenchymal type cells. The mesenchymal type cells, in contrast, demonstrated higher AC133, CD73, CD105, CD117, EGFR, integrin α_2_β_1_, and CD146 surface marker expression than the epithelial type cells.

**Conclusion:**

The established culture system provides an in vitro model for the selection of drugs that target cancer-associated stromal progenitor cells, and for the development of ovarian cancer treatments.

## Background

Ovarian cancer is the fifth leading cause of death from cancer in the Western world, and the leading cause of death from gynecologic cancer. More than 90% of ovarian cancers arise from the surface epithelium [1,2]. In Taiwan ovarian cancer is the tenth leading cause of female malignancy and the leading cause of death from gynecological cancer. Seventy-five percent of epithelial ovarian cancer (EOC) patients receive a diagnosis at the advanced stage, and among these, at least one-third are associated with the development of ascites, an abnormal collection of exudate with a cellular fraction consisting mainly of cancer cells, lymphocytes, and mesothelial cells [3,4]. Efforts at improving the survival of patients with EOC have focused on early detection and on the development of novel chemotherapeutic drugs. However, long-term survival of patients with advanced ovarian cancer remains limited (below 20%). Understanding the mechanisms underlying the initiation and progression of ovarian cancer is therefore essential for the development of effective treatments.

Components of ascites, including neoplastic cells and pro-angiogenic or tumorgenic factors, may contribute to the proliferation and spread of cancer cells [5]. Results from previous investigations indicate that in addition to the neoplastic cancer cells, stromal cells that are heterogeneous and composed of fibroblasts, endothelial or methothelial cells, adipocytes or adipose tissue-derived stromal cells, bone marrow-derived stem cells, and immune cells promote tumor growth, invasion, and metastasis by cross-talk with cancerous cells [6,7]. Prior research has established that ascites commonly develops in patients with EOC, and that the presence of abnormal stromal cells in the ascites may establish an unusual microenvironment for tumor spreading. However, the roles of these abnormal stromal cells in the development of ovarian cancer remain poorly understood.

Tumor development is mainly associated with the accumulation of multiple genetic and epigenetic alterations, resulting in the transformation of a normal cell to a cancer cell [8]. The components of tumors are complex, comprising genetically or epigenetically altered tumor cells surrounded by a heterogeneous population of stromal cells. The cellular and molecular interactions between tumor and stromal cells trigger tumor growth and metastatic spreading [9]. Increasing evidence has suggested that the growth capability of a tumor is dependent on cancer stem cells or cancer initiating cells (CSCs/CICs), which represent a minority of cells within tumors [10]. Although investigators proposed the existence of CSCs/CICs several decades ago, it was not until 1997 that Bonnet et al. first isolated these cells from patients with acute myeloid leukemia [11]. Further studies subsequently described the isolation of CICs from patients with prostate, melanoma, lung, colon, and pancreas cancer [12-16]. CICs may be derived from abnormal stem cells or from differentiated tumor cells acquiring stem-like characteristics.

The epithelial origin of most ovarian cancers and co-expression of epithelial and mesenchymal markers (keratin and vimentin or N-cadherin, respectively) in EOC highlight the importance of phenotypic and genetic plasticity of the ovarian surface epithelium (OSE) during neoplastic transformation and acquisition of stem cell characteristics [17]. Recent studies have shown that epithelial mesenchymal transition (EMT), which mediates changes in cell morphology and involves the loss of cell adhesion and acquisition of migratory and invasive properties through an undefined mechanism, triggers the generation of CICs from immortalized human mammary epithelia [18-20]. Disruptions to the normal barrier, which protects normal OSE from underlying stromal signaling, may also promote EMT, resulting in neoplastic transformation of the OSE [21]. Hanahan and Weinberg detected several molecular changes during EMT, including the loss of epithelial markers (E-cadherin) and the induction of mesenchymal markers (N-cadherin, fibronectin, and Snail) [8], and implicated these changes in tumor recurrence. Stromal progenitor cells from the ascites of patients with ovarian cancer have been shown to promote the cancer tumorigenecity and angiogenesis [22]. We thus aimed to isolate and characterize stromal progenitor cells from the ascites of patients with epithelial ovarian adenocarcinoma (EOA), applying various in vitro culture conditions to obtain 2 distinct cell populations with different morphologies, the epithelial and mesenchymal types, and comparing them with the cells isolated from fresh cancer tissues of EOA patients. The isolated cells with epithelial morphology and epithelial-specific marker expression were more frequently from the ascites of patients with early stage EOA, whereas mesenchymal type cells were derived from the ascites of the same patient with late stage EOA and recurrent tumors. During the in vitro culture process, these cells displayed the potential for self-renewal and presented with EMT capability.

## Methods

### Ascites collection from patients with epithelial ovarian adenocarcinoma

The study protocol was approved by the Institutional Review Board of Cathay General Hospital. Seventeen patients with primary or recurrent epithelial ovarian cancer were recruited. Seventeen ascitic fluid samples and 7 fresh tissue samples were obtained from 16 EOA patients, with ages ranging from 28 to 85 years, attending Cathay General Hospital between May 2009 and January 2010. Two normal ovarian tissue samples were obtained from histologically proven normal ovaries of 2 patients with early ovarian cancer. Histopathology, grade, and stage of ovarian tumor were assigned according to the Federation of Gynecology and Obstetrics criteria. Of the 17 ascitic fluid samples included, 12 were from patients diagnosed with ovarian serous adenocarcinoma (OSA), 3 were from patients with ovarian clear cell adenocarcinoma (OCCA), and 2 were from patients with ovarian mucinous borderline tumor (OMBT). Table 1 shows details of the clinical characteristics of the 16 patients from which the 17 ascitic fluid samples and 7 fresh tissues samples were obtained. An average of 20 mL ascites was harvested at laparotomy or ultrasound-guided paracentesis was centrifuged to fractionate the cellular component.

**Table 1:** Clinical characteristics of patients from which ascites were obtained and characteristic features of cells isolated

Ascites Sample No.	Age	Histopathology	Origin	Stage	Prior CT	Clinical intervention	Cell Morphology	Percentage of cells with CK-18 (+)/Vimentin (+)
1	85	OSA	ascites	IIIC	yes	Neoadjuvant CT	Mesenchymal-like	6.5%/95.3%

2	54	OSA	ascites	IIIC	yes	Recurrence	Epithelial-like	73.1%/11.2%

3	45	OSA	ascites	IIIC	yes	Recurrence	Epithelial-like	79.8%/8.5%

4	62	OSA	ascites	IIIC	No	Primary surgery	Mesenchymal-like	25.1%/85.5%
							
							Epithelial-like	79.5%/9.2%

5	62	OSA	ascites	IC	No	Primary surgery	Mesenchymal-like	21.3%/90.4%

6	57	OSA	ascites, tissue	IIC	No	Primary surgery	Mesenchymal-like	15.3%/92.7%
								
								4.8%/92.3%

7	46	OSA	ascites	IIIC	Yes	Recurrence	Mesenchymal-like	21.4%/87.2%

8	48	OSA	ascites	IIIC	No	Primary surgery	Mesenchymal-like	15.9%/91.5%
								
			tissue					4.7%/97.5%

9	53	OSA	ascites	IIIC	No	Primary surgery	Mesenchymal-like	24.3%/91.7%
								
			tissue					3.5%/98.1%

10	48	OSA	ascites	IIIC	No	Primary surgery	Mesenchymal-like	13.9%/94.5%
								
			tissue					5.5%/94.8%

11	28	OMBT	ascites	IA	No	Primary surgery	Mesenchymal-like	4.7%/98.9%

12(3)	45	OSA	ascites	IIIC	yes	Recurrence	Mesenchymal-like	5.4%/93.5%

13(7)	46	OSA	ascites	IIIC	Yes	Recurrence	Mesenchymal-like	1.3%/99.4%

14(11)	28	OMBT	ascites	IA	Yes	Recurrence	Mesenchymal-like	4.2%/98.7%

15	37	OCCA	ascites	IC	Yes	Recurrence	Mesenchymal-like	1.4%/97.1%

16	52	OCCA	ascites	IC	Yes	Secondary debulking	Mesenchymal-like	6.2%/91.8%

17	71	OCCA	ascites, tissue	IIIC	No	Primary surgery	Mesenchymal-like	8.2%/93.1%
								
								3.2%/95.7%

18	61	OSA	tissue	IIIC	no	primary surgery	Mesenchymal-like	4.3%/96.1%

19	73	OSA	tissue	IIIC	no	primary surgery	Mesenchymal-like	7.1%/92.5%

20	N/A	Endometriosis	ascites	N/A	N/A	N/A	Mesenchymal-like	3.2%/97.8%
							
							Epithelial-like	99.1%/3.4%

( ): Sample obtained from the same patient No.

OSA: ovarian serous adenocarcinoma; OCCA: ovarian clear cell adenocarcinoma; OMBT: ovarian mucinous borderline tumor

CT: chemotherapy

N/A: not applicable

### Isolation and in vitro culture conditions of ascitic cells

Cells from ascites samples of patients diagnosed with malignant EOA were isolated using standard procedures. Briefly, the ascitic fluid was centrifuged at room temperature for 5 min at 1500 rpm. The cell pellet was resuspended in 10-mL ovarian culture medium [basal medium A: DMEM/F12 medium supplemented with 10% FBS, EGF (10 ng/mL) and FGF-b1 (10 ng/mL) or basal medium B: M199 medium supplemented with 10% FBS, EGF (20 ng/mL) and hydrocortisone (0.4 μg/mL)] for every 3 × 10^6 ^cells in a T75 flask. Cultures were maintained in a humidified chamber with 5% CO_2 _at 37°C, and the media were refreshed every 3 days to maintain the adherent cells. When the adhered cells reached 85% confluence, the cells were harvested with 0.25% trypsin - 1 mM EDTA (Sigma-Aldrich, St. Louis, MO, USA) treatment for 5 min.

### Flow cytometric analysis

Expanded cells from ascites of patients with EOA were characterized in vitro using flow cytometry (FACSCalibur, BD Biosciences, USA). Fluorescein isothiocyanate (FITC)- or phycoerythrin (PE)-conjugated antibodies against CD24 (BioLegend, CA, USA), CD44 (BioLegend), AC133 (Miltenyi Biotec, CA, USA), CD73 (BD, CA, USA), CD105 (Pharmingen), CD117 (eBioscience, CA, USA), cytokeratin 18 (Dako), CXCR4 (Pharmingen), EGFR (BioLegend), PDGFR (Pharmingen), intergrin α_2_β_1 _(Abcam, CA, USA), CD146 (Abcam), FLT-4 (BioLegend), and E-cadherin (BioLegend) were used.

### Immunofluorescence analysis of markers for mesenchymal and epithelial cells

Immunofluorescence analysis was performed on cells in vitro at the third passage. Antibody against vimentin (the mesenchymal marker (Dako); 1:250 dilution) or against cytokeratin 18 (the epithelial marker (Dako); 1:250 dilution) were used, followed by FITC-conjugated secondary antibody, and observation under a fluorescence microscope. DAPI staining was used to localize the nuclei.

### Cell growth kinetics of isolated ascites cells

Isolated ascites cells were initially seeded in vitro at a density of 10^5 ^cells in a T75 flask with 10 mL of complete medium. Cells were harvested in log-phase growth, at approximately 85% confluence using Trypsin-EDTA (Sigma, T4174). The cell number was counted using a hemocytometer. The growth fraction (GF) was defined as the average growth rate in between each 2 passages. The cell doubling time was calculated by least squares regression analysis of a semi-logarithmic plot.

### Isolation of normal ovarian and ovarian carcinoma tissue cells

Normal ovarian and ovarian carcinoma tissues were minced in Hank's balanced salt solution (Invitrogen; Grand Island, NY) and mixed with 1 mg/ml of collagenase 1A (Sigma, C9891) at 37°C for 60 minutes. The dispersed cells were filtered through a 70 ?m nylon mesh (BD Bioscience) to remove the undigested tissue pieces, and further centrifuged at 170 xg to obtain the cell pellet. Isolated cells were initially seeded in vitro at a density of 5 × 10^4 ^cells per cm^2 ^in a T75 flask. Cells were sub-cultured in log-phase growth, at approximately 80% confluence using Trypsin-EDTA (Sigma, T4174).

## Results

### Cells deriving from ascites of patients with EOA expressed mesenchymal or epithelial cell-specific surface markers

**Figure 1:**
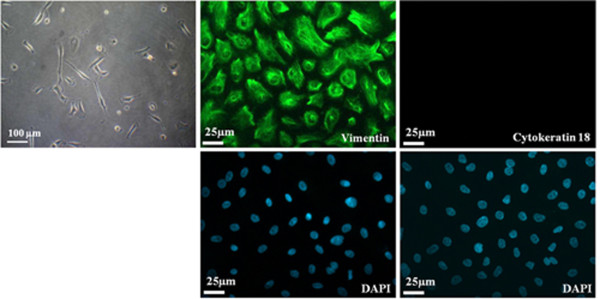
**Immunostaining of ascites cells of a patient with ovarian adenocarcinoma (#13)**. Images (400×) of the ascites cells (passage 3, cultured in medium B) from a patient with ovarian adenocarcinoma (#13), immunostained with antibodies against vimentin (mesenchymal cell marker, green) or cytokeratin 18 (epithelial cell marker, green). DAPI (blue) was used to localize nuclei. A representative phase-contrast image (100×) is also shown.

**Figure 2:**
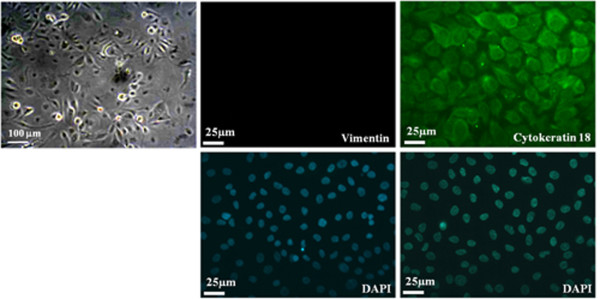
**Immunostaining of ascites cells of a patient with ovarian adenocarcinoma (#2)**. Images (400×) of the ascites cells (passage 3, cultured in medium B) from a patient with ovarian adenocarcinoma (#2), immunostained with antibodies against vimentin (mesenchymal cell marker, green) or cytokeratin 18 (epithelial cell marker, green). DAPI (blue) was used to localize nuclei. A representative phase-contrast image (100×) is also shown.

**Figure 3:**
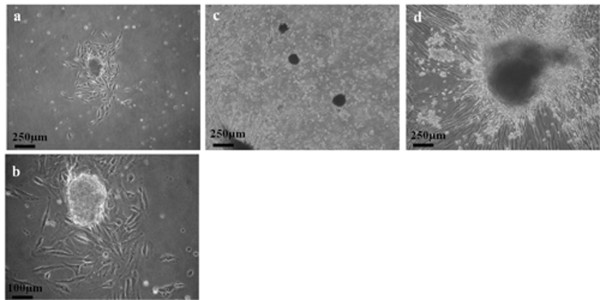
**Morphology of spheroid tumor stromal progenitor cells from fresh ovarian cancer tissues**. Phase contrast images of ovarian cancer cells (passage 2, cultured in medium B) deriving from patients with serous type adenocarcinoma: #6 (a: 40X; b: 100X), #8 (c: 40X), and #9 (d: 40X). All cells display the mesenchymal-like morphology of the ascites cells from the same patient, forming spheroid-like structures in most observed fields.

**Figure 4:**
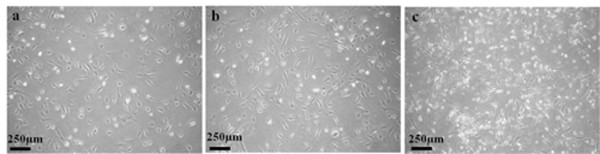
**Morphology of cells deriving from normal human ovarian tissue**. Phase contrast images of cells (passage 2, cultured in medium B) deriving from normal ovarian tissue of patients #1 (a: 40X) and #2 (b: 40X). Both display the mesenchymal-like morphology of the cancer cells deriving from cancer tissue of patient #6 (c: 40X).

The present study isolated two different morphological cell populations from EOA patients' ascites, culturing in selective media. One population of cells was cobblestone shaped, demonstrating epithelial-like morphology. The other was spindle shaped, demonstrating mesenchymal-like cell morphology (Figures 1 and 2, photos of phase-contrast microscopic observations). Analysis of immuno-fluorescence staining revealed that the cells with mesenchymal type morphology expressed vimentin, the mesenchymal cell-specific marker, but not cytokeratin 18, which is specific to epithelial cells (Figure 1). In contrast, cells with epithelial morphology expressed cytokeratin 18, but not vimentin, in the cytosol (Figure 2). Cells sprouting from the fresh ovarian cancer tissue or normal ovarian tissue samples showed mesenchymal-like morphology (Figures 3 and 4). In vitro culturing of EOA patient tissue produced a spheroid-like formation of spindle-shaped cells, suggesting the presence of tumor stromal progenitors (Figure 3). Similar observations occurred in cells cultured from the ascites of the same patient (data not shown), indicating that the mesenchymal type cells identified may originate from the tumor stroma.

**Figure 5:**
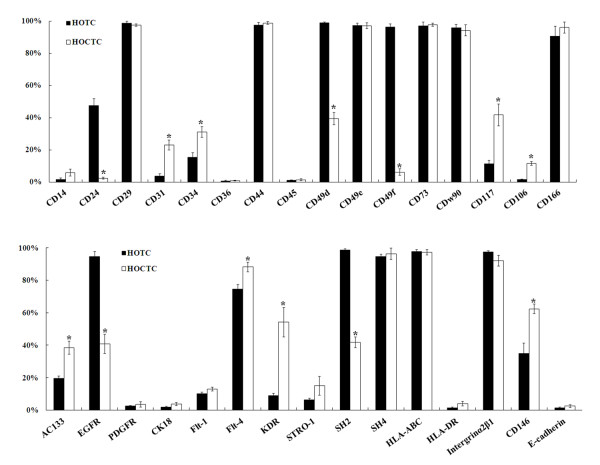
**Expression of surface markers on cells deriving from OSA tissues and normal ovarian tissues**. Marker expression on the surfaces of cells (cultured in medium B) deriving from ovarian serous type adenocarcinoma tissues, analyzed using flow cytometry with various antibodies. HOTC: mesenchymal-like cells deriving from normal ovarian tissue (n = 3). HOCTC: human ovarian cancer tissue cells (stage IIIc, n = 3). CK18 = cytokeratin 18. *Denotes a significant difference (P < 0.05) compared with HOTC samples analyzed by Student's t-test.

**Figure 6:**
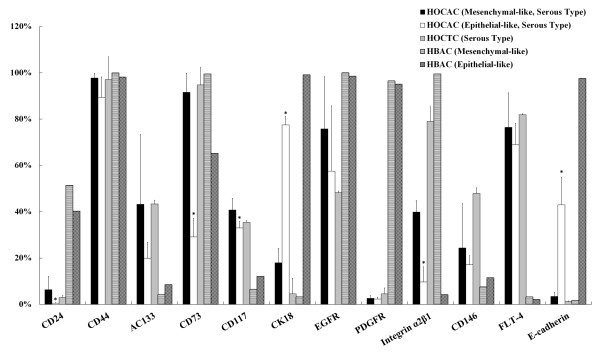
**Expression of surface markers on cells deriving from ascites and OSA tissues of patients, and ascites of endometriosis patient**. Flow cytometric analysis of surface marker expression by the mesenchymal-like (n = 8) or epithelial-like cells (n = 3) (cultured in medium B) deriving from the ascites of patients with ovarian adenocarcinoma (HOCAC; stage IIIc) and cells (cultured in medium B) (HOCTC) deriving from cancer tissues of patients with ovarian adenocarcinoma (stage IIIc, n = 3); and cells deriving from benign ascites of patients with endometriosis (HBAC). *Denotes a significant difference (P < 0.05) compared with HOTC samples analyzed by Student's t-test.

**Figure 7:**
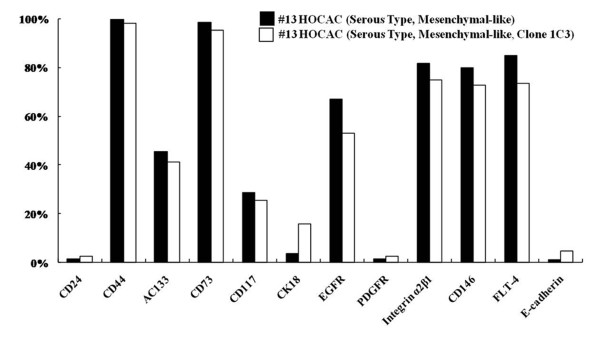
**Expression of surface markers on single-cell clones and the original mesenchymal-like cells**. Flow cytometric analysis of surface marker expression by cells (passage 6, cultured in medium B) of single-cell clone 1C3 deriving from the mesenchymal-like cell population isolated from the ascites of a patient with OSA (#13) and the original mother cells (passage 6, cultured in medium B).

Flow cytometric analysis using antibodies against cell surface molecules further characterized the ascites- or tumor tissue-derived cells. Results showed that the cells with mesenchymal-like morphology deriving from the ovarian cancer tissues of patients with OSA had higher CD31, CD117, CD106, AC133, and CD146 expression and moderately increased CD34 and Flt-4 expression, but lower CD24, CD49d, CD49f, EGFR, and SH2 expression, compared to cells deriving from normal ovarian tissue (Figure 5). Cells with mesenchymal-like morphology deriving from normal and cancer tissues showed similar levels of CD14, CD29, CD36, CD44, CD45, CD49e, CD73, CDw90, CK18, FLT-1, STRO-1, SH4, HLA-ABC, and integrin α_2_β_1 _expression (Figure 5). Cells with mesenchymal- or epithelial-like morphology deriving from the ascites of patients with OSA expressed similar levels of CD44, AC133, CD146 and Flt-4 (Figure 6). CD24, CD73, CD117 and integrin α_2_β_1 _expressions were higher in the mesenchymal-like cells deriving from ascites or cancer tissues than the normal HOTC, but lower in epithelial-like cells from ascites. The epithelial-like cells, in contrast, demonstrated higher cytokeratin 18 and E-cadherin expression (Figure 6). Expressions of CD24 and PDGFR were reduced in both epithelial- and mesenchymal-like cells deriving from ovarian ascites (HOCAC) than those from normal ascites (HBAC). Epithelial- and mesenchymal-like cancer cells deriving from ascites both demonstrated the typical cancer stem/progenitor cell characteristics of CD44^high^, CD24^low^, and AC133^+ ^as reported in ovarian cancer-initiating cells by Zhang et al., and ovarian cancer stem cells by Alvero et al. [23,24]. The mesenchymal-type identity was further confirmed by specific surface molecule expressions in the single-cell-derived clones (Figure 7).

### Growth curves of ascites- or tumor tissue-derived cell populations

**Figure 8:**
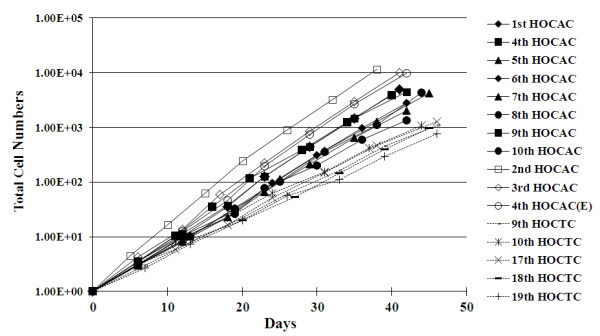
**Growth curves of cells deriving from ovarian adenocarcinoma ascites and tissue**. The doubling time of mesenchymal-like ascites cells (#1, 4, 5, 6, 7, 8, 9, and 10) was approximately 44.2 hrs (n = 8, s = 6.2). The doubling time of epithelial-like ascites cells [#2, 3, and 4 (E)] was approximately 36.3 hrs (n = 3, s = 3.9). The doubling time of mesenchymal-like cells from ovarian tissue (#9, 10, 17, 18, and 19) was approximately 56.3 hrs (n = 5, s = 4).

Analysis of the growth characteristics of 2 cell populations with distinct morphology deriving from ascites of different ovarian adenocarcinoma samples over a 5-week in vitro culturing period revealed that the epithelial type cells grew faster than the mesenchymal type cells. The doubling time of the epithelial type cells was 36 h, compared to a doubling time of 44 h in the mesenchymal type cells deriving from ascites, and a doubling time of 56 h in cells from fresh cancer tissues (Figure 8). Both cell populations could be cultured long-term in vitro for more than 2 months (Figure 8).

## Discussion

Current study describes two different types of tumors cells with distinct mesenchymal-like and epithelial-like morphologies and their specific markers, which were derived from the ascites and tumor tissues of patients with epithelial ovarian cancer. Both cell populations were selected and cultured from the same adherent cells. The mesenchymal type cells expressed vimentin (a mesenchymal cell marker), whereas the epithelial type cells expressed cytokeratin 18 (an epithelial cell marker), as revealed by immunocytochemical and flow cytometric analyses (Figures 1, 2, and 6). The mesenchymal-like cells demonstrated higher CD73, CD117, and integrin α_2_β_1 _surface marker expression than the epithelial-like cells (Figure 6). These cells surrounded the aggregated or spheroid tumor mass (Figure 3), suggesting that this mesenchymal-like cell population may originate from tumor stroma.

Downregulation of E-cadherin is a hallmark of the epithelial mesenchymal transition (EMT). EMT progression in cancer cells is associated with the loss of certain epithelial markers and the acquisition of a mesenchymal phenotype, as well as migratory activities [18-20]. The characteristics of both types of cells isolated in this study resembled those of stem/progenitor cells for their potential for self-renewal and expression of typical cancer stem/progenitor cell markers [25-28], including CD44^high^, CD24^low^, and AC133^+ ^(Figures 7 and 8). These two cell populations could be cultured long-term in vitro for more than 2 months (Figure 8), and could be cloned without losing their original identities (Figure 7). Future investigative aims include the evaluation of the tumorigenecity and metastatic ability of these two cell populations in nude mice to further understand their characteristics.

Tumors comprise neoplastic and stromal cell components. Previous research has established that interactions between cancer and stroma are important for cancer progression. Stromal cell function reportedly plays a pivotal role during ovarian cancer tumorigenesis: infiltration of endothelial cells into ovarian carcinoma tumors is dependent on the presence of myofibroblasts [29] and stromal infiltration, as well as vascular maturation, which together function as a checkpoint linking angiogenesis with the initiation of tumor progression [30]. Several studies have demonstrated that ovarian cancer cells were able to attach to peritoneal mesothelial cells by the activation of CD44 or integrin α_2_β_1 _[31,32]. In the study by Burleson et al., ascites spheroids (multi-cellular aggregates) of ovarian carcinoma were able to adhere to live, but non-fixed, human mesothelial cells [33,34]. Recent analysis using surgical specimens suggested that mesothelial cells may nurture peritoneal metastases through the production of growth factors such as vascular endothelial growth factor (VEGF) and fibroblast growth factor 2 (FGF2) [35], confirming Wilson's finding that mesothelial cells from ovarian cancer patients were able to stimulate the clonogenic growth of ovarian tumor cells [36]. The mutual expression of highly tumorgenic and metastatic-related genes, such as CD44, integrin α_2_β_1_, CD146, EGFR, and Flt-4, by epithelial- and mesenchymal-like cells indicates that cancer-stromal interactions of both types of cells may occur through common signaling pathways (Figure 6). In compare to the mesenchymal stromal cells (HOTC) of normal ovarian tissue, we have shown that mesenchymal like cancer tumor fibroblasts (HOCTC) sprouting from ovarian cancer tissue expressed higher levels of BMP2, BMP4 and TGF-β, which are highly associated with tumorigenesis and metastasis. These cells also had higher expressions of angiogenic-related markers (e.g. KDR, Flt-4, and CD31), tumorigenic molecules (CD24^low^/CD44^high^, AC133), progenitor characteristic markers (CD117, CD34, CD14, and CD146) and a diminished expression of mesenchymal cell adhesion molecules (e.g. CD24, CD49d, CD49f, SH2, and SH4) (Figure 5). These results are consistent with the characteristics of stromal (mesenchymal) stem cells in human ovarian tumor microenvironment (CA-MSCs) recently reported by McLean et al. [37], suggesting that HOCTC may be able of regulating cancer stem cells and promoting tumorigenesis via altered BMP expression, as the CA-MSCs.

Martinet et al. recently isolated hospicells (ascites-deriving stromal cells) from the ascites of FIGO stage III ovarian cancer patients [38]. These could promote tumorigenicity and angiogenesis by increasing HIF1 and VEGF expression [22]. In the present study, the molecular markers of progenitors in cells isolated from ascites and tumor tissues of patients with advanced or recurrent ovarian cancer were the highly expressed EGFR and Flt-4 (VEGF receptor) (Figure 6), which are important during tumor angiogenesis and metastasis. These results agree with reported findings in hospicells [22].

To our knowledge, this is the first study to report the isolation of cells with different morphologies, epithelial type, and mesenchymal type, from the ascites and tumor tissues of patients with epithelial ovarian adenocarcinomas, which could represent the stem/progenitor cells in ovarian adenocarcinoma. These ovarian adenocarcinoma stem/progenitor cells may possess the ability for EMT, as indicated by the presence of specific molecular markers, which may function to program this process. The established in vitro culture system provides an ideal model for further investigation of drugs that target cancer-associated stromal/progenitor cells, and for the development of effective ovarian cancer treatments. Ultimately, understanding the complex molecular networks among stromal cells and tumor cells will provide useful information for the therapeutic targeting of human cancers.

## Conclusions

The present study established and validated an in vitro culture system for the future screening of drugs targeting the ovarian cancer-associated stromal progenitor cells, providing an alternative tool for the development of effective ovarian cancer therapy. The obtained ascites-originating cells with epithelial morphology and epithelial-specific markers more frequently derived from EOA patients at an earlier stage; mesenchymal type cells mostly derived from EOA patients at late stage and recurrent tumors. Both cell populations displayed the potential for self-renewal and EMT capabilities during the in vitro culture process.

## Competing interests

The authors declare that they have no competing interests.

## Contributions

SFC and DTBS contributed equally and are corresponding authors of this paper. CMH and TYC participated in collection and clinically characterization of all samples; CCH performed cell isolation and most of the analyses; CCH, SFC and DTBS analyzed data; CMH, SFC and DTBS designed most of the experiments, participated in writing the paper; SFC and DTBS conceived and coordinated the study. All authors read and approved the final manuscript.
